# Pathological Features Associated with Lymph Node Disease in Patients with Appendiceal Neuroendocrine Tumors

**DOI:** 10.3390/cancers16162922

**Published:** 2024-08-22

**Authors:** Salvador Rodriguez Franco, Sumaya Abdul Ghaffar, Ying Jin, Reed Weiss, Mona Hamermesh, Andrii Khomiak, Toshitaka Sugawara, Oskar Franklin, Alexis D. Leal, Christopher H. Lieu, Richard D. Schulick, Marco Del Chiaro, Steven Ahrendt, Martin D. McCarter, Ana L. Gleisner

**Affiliations:** 1 Division of Surgical Oncology, Department of Surgery, University of Colorado, Anschutz Medical Campus, Aurora, CO 80045, USA; 2 Department of Biostatistics and Informatics, University of Colorado, Anschutz Medical Campus, Aurora, CO 80045, USA; 3 Division of Medical Oncology, School of Medicine, University of Colorado, Anschutz Medical Campus, Aurora, CO 80045, USA; 4 Cancer Center, University of Colorado, Anschutz Medical Campus, Aurora, CO 80045, USA

**Keywords:** neuroendocrine tumors, nodal disease risk, predicted risk, practice patterns

## Abstract

**Simple Summary:**

This study investigated whether certain features of appendiceal neuroendocrine tumors, beyond tumor size, can help predict the risk of lymph node invasion. We reviewed over 5000 cases from a national cancer database and found that in addition to the size of the tumor, lymphovascular invasion is a very strong risk predictor. Interestingly, we discovered that the growth of the tumor into deeper layers, which is usually considered a critical factor in evaluating small tumors, did not significantly affect the risk of lymph node invasion. This study aims to refine how we assess the risk of local spreading, encouraging a more tailored surgical approach, which could prevent unnecessary procedures and better focus on what is truly necessary for patient care.

**Abstract:**

This study aimed to evaluate the role of pathological features beyond tumor size in the risk of lymph node metastasis in appendiceal neuroendocrine tumors. Analyzing data from the national cancer database, we found that among 5353 cases, 18.8% had lymph node metastasis. Focusing on tumors smaller than 2 cm, a subject of considerable debate in treatment strategies, we identified lymphovascular invasion as one of the strongest predictors of lymph node disease. Interestingly, extension into the subserosa and beyond, a current factor in the staging system, was not a strong predictor. These findings suggest that careful interpretation of pathological features is needed when selecting therapeutic approaches using current staging systems.

## 1. Introduction

Appendiceal neuroendocrine tumors (ANETs) are the most common malignant appendiceal neoplasm, incidentally found in approximately 0.5% of all appendectomies [1,2,3]. Epidemiological studies in both the United States and Europe reveal a consistent incidence rate, ranging from 0.08 to 0.2 cases per 100,000 individuals annually [4,5,6].

Localized ANETs are typically managed with an appendectomy [7]; however, a right colectomy may be necessary to achieve negative margins or in patients with a high risk of nodal disease [3,8,9]. Tumor size is a well-established risk factor and has traditionally guided the extent of surgical intervention and surveillance strategies; for example, tumors measuring ≥ 2 cm warrant a right colectomy in all current guidelines [2,3,8,10,11]. Although smaller tumors (<2 cm) generally present a low risk of regional and metastatic disease, this risk is not negligible [12]. Consequently, in this setting, other pathologic features, such as tumor depth, tumor differentiation, and the presence of lymphovascular invasion (LVI) have been proposed to inform the extent of surgical treatment [2,8,10].

Building on this, previous studies have shown that LVI increases the odds of nodal disease by 3.4 to 10 [13,14]. Similarly, invasion into the subserosa and beyond has also been shown to increase the odds of nodal disease by 1.7 to 3.6 [14]. Tumors with these features are currently classified as T3 tumors regardless of their size, according to the AJCC staging [15]. However, the prognostic value of these pathologic features remains controversial, particularly in patients for whom the necessity of a right colectomy is debated.

In the current study, we aimed to determine if high-risk pathologic features for regional disease in appendiceal NET can help inform surgical treatment for patients with tumors that are less than 2 cm. Specifically, we sought to determine if the association between the pathologic features (tumor depth and LVI) and nodal disease varies according to tumor size. Additionally, we used our findings to estimate the predicted probability of nodal disease in patients with ANETs according to the size and the presence of high-risk pathologic features.

## 2. Materials and Methods

Data sources and patient selection. This retrospective analysis utilized data from patients diagnosed with ANETs between 2004 and 2021 included in the National Cancer Data Base (NCDB). The NCDB is a joint project of the Commission on Cancer of the American College of Surgeons and the American Cancer Society, capturing approximately 70% of all new cancer diagnoses in the United States and Puerto Rico through over 1500 Commission on Cancer-accredited facilities. The Colorado Multiple Institutional Review Board (COMIRB) granted an exemption for this study given the utilization of de-identified data.

**Figure 1:**
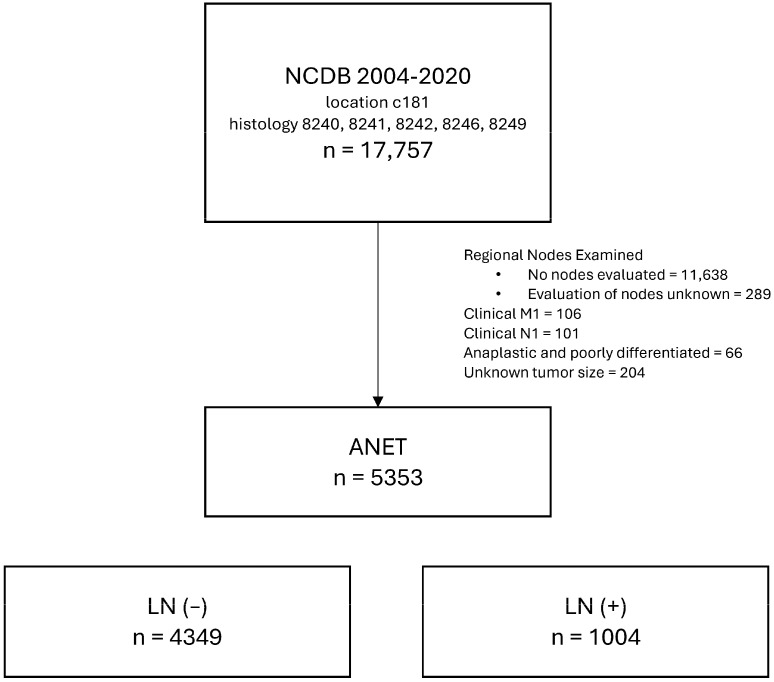
CONSORT diagram showing the cohort selection process.

Inclusion criteria and histological classification. Eligible cases were identified using morphology codes from the International Classification of Diseases for Oncology, 3rd Edition (ICD-O-3), indicative of neuroendocrine tumors (8240, 8241, 8242, 8246, 8249). Eligibility required at least one lymph node removed/evaluated during surgery. Cases were excluded if there was evidence of clinical node disease at diagnosis, clinical metastatic disease at diagnosis, anaplastic cases, no data regarding tumor size, or lymph node examination results were missing or unknown. Selection criteria are summarized in Figure 1.

Patient demographics and baseline characteristics. Patient demographics and baseline characteristics were categorized as follows: age (≤25, 26–50, 51–75, ≥75 years), sex (male, female), race (Non-Hispanic White, Non-Hispanic Black, Non-Hispanic Asian, Non-Hispanic, Hispanic, Other), Charlson–Deyo Comorbidity Index (0–1, ≥2), and insurance status (Medicaid/Medicare/other government, private insurance, uninsured, unknown).

Pathological features. Pathological features assessed included tumor differentiation (well differentiated, moderately differentiated), LVI (absent, present, unknown), depth of tumor extension (confined to the appendiceal wall [limited to the mucosa, submucosa, or muscularis propria], invading the subserosa and beyond, unknown), tumor size (<1 cm, 1 to <2 cm, ≥2 cm), surgical margins (negative, positive, unknown), and lymph node disease (N—[zero positive nodes], N + [one or more positive nodes]).

Statistical analysis. Statistical analyses were conducted using STATA version 18.0 (StataCorp, College Station, TX, USA). Descriptive statistics were used to summarize categorical variables (frequencies, percentages). Unadjusted and adjusted binomial logistic regression models, along with their respective receiver operating characteristic (ROC) curves and areas under the curve (AUCs), were used to identify significant predictors of lymph node disease. In order to validate our results against previous publications, we performed sensitivity analyses using the same variables as in the adjusted binomial logistic model. Sensitivity analyses included a complete case analysis (cases with no missing data), an analysis focused on histological codes 8240 (carcinoid tumor) and 8241 (enterochromaffin cell carcinoid), one including only patients with at least 12 evaluated nodes, one combining these three characteristics, and another evaluating the depth of tumor in more than two categories.

We also looked for the possible presence of effect modification between tumor size, depth of tumor, and presence of LVI using a logistic regression model including two- and three-way interaction terms. Additionally, we used marginal analysis to calculate the predicted probability of lymph node disease for different combinations of LVI and depth of tumor across different tumor sizes. Significant results were set at a two-sided alpha of 0.05.

## 3. Results

Out of the 5353 patients with appendiceal neuroendocrine tumors included in our study, 1004 (18.8%) had nodal disease (N+). The majority of patients were female (61.1%), aged 51–75 years (42.7%), and identified as Non-Hispanic White (81.5%). Most patients were insured under Medicaid, Medicare, or other government plans (58.5%), and a significant proportion were treated at Academic/Research Programs (21.5%) and Integrated Network Cancer Program (14.8%). Regarding tumor size, most patients had smaller tumors, <1 cm (45.8%). A comprehensive breakdown of additional demographics and clinical characteristics is presented in Table 1.

### 3.1. Factors Associated with Nodal Disease

On univariate analysis, several factors were associated with nodal disease. Female patients showed a higher likelihood of nodal disease than males (OR 1.19 [95% CI 1.03–1.37]). Non-Hispanic Black patients had a higher risk than Non-Hispanic White patients (OR 1.61 [95% CI 1.29–2.01]). Age groups 51–75 and ≥76 years showed a reduced risk compared to the ≤25 age group (0.56 [95% CI 0.46–0.68] and 0.29 [95% CI 0.20–0.42], respectively). Similarly, Charlson–Deyo scores ≥2 and those insured under Medicaid/Medicare or other government programs showed a reduced risk of nodal disease (OR 0.44 [95% CI 0.31–0.63] and OR 0.54 [95% CI 0.46–0.63], respectively). In addition, pathological characteristics were associated with nodal disease. Moderately differentiated tumors (OR 2.07 [95% CI 1.67–2.61]), presence of lymphovascular invasion (OR 7.43 [95% CI 6.23–8.86]), and tumor size ≥1 cm but <2 cm, and ≥2 cm had increased risk of nodal disease (OR 6.21 [95% CI 4.8–8.01] and 21.45 [95% CI 16.9–27.2], respectively). Tumor depth, specifically invading the subserosa and beyond, was not associated with nodal disease (OR 1.01 [95% CI 0.82–1.23]).

After adjusting for patient and tumor characteristics, factors independently associated with nodal disease included age (51–75 years, aOR 0.68 [95% CI 0.54–0.87]; ≥76 years, aOR 0.54 [95% CI 0.35–0.85], compared to patients 25 years old or less), insurance status (Medicare/Medicaid/OG, aOR 0.78 [95% CI 0.64–0.95], compared to patients with private insurance), LVI (aOR 4.09 [95% 3.37–4.97]), and tumor size (≥1 cm but <2 cm, aOR 4.16 [95% CI 3.18–5.43]; ≥2 cm, aOR 14.43 [95% CI 11.27–18.49], compared to patients with tumors less than 1 cm). Tumor size and LVI were the strongest predictors of nodal disease (AUC 0.70 and 0.79, respectively). Univariate and multivariate models are presented in Table 2. Similar results were observed in the sensitivity analyses, where LVI and tumor size consistently remained the strongest independent predictors of nodal disease. Sensitivity analyses are depicted in Tables S1 and S2.

### 3.2. Effect Modification Analysis

In analyzing the possible effect modification between pathologic features currently considered high-risk (tumor depth, LVI, and tumor size), subserosal invasion and beyond was not associated with nodal disease at any tumor size. Among patients with tumors <1 cm, the aOR for nodal disease with subserosal invasion was 1.39 (95% CI 0.50–3.88) in the absence of LVI; similarly for tumors ≥1 cm but <2 cm, aOR was 0.73 (95% CI 0.41–1.28). Conversely, LVI was strongly associated with nodal disease across all tumor sizes with similar associations regardless of the presence of subserosal invasion. For instance, in tumors <1 cm, LVI was associated with an aOR of 10.1 (95% CI 2.71–37.84) in patients with no subserosal invasion and 14.1 (95% CI 4.85–41.24) in patients with subserosal invasion. In tumors ≥1 cm but <2 cm, LVI was associated with an aOR 5.86 (95% CI 2.69–12.79) when tumors were confined to the appendiceal wall and 3.07 (95% CI 1.74–5.42) in tumors with invasion of the subserosa and beyond. Effect modification analyses are summarized in Table 3 and fully described in Supplementary Table S3.

### 3.3. Probability of Nodal Disease

Predicted probabilities of at least one positive lymph node were calculated according to the presence or absence of the different pathologic features, according to tumor size, and were compared with the observed rates. For tumors smaller than 1 cm, the predicted probability of nodal disease was very low unless LVI was present (confined to the appendiceal wall, LVI absent 2.2% vs. LVI present 18.1%; invading the subserosa and beyond, LVI absent 2.9% vs. LVI present 23.4%). Regarding tumors ≥1 cm but <2 cm, the same patterns were observed (confined to the appendiceal wall, LVI absent 13.9% vs. LVI present 47.8%, invading the subserosa and beyond, LVI absent 10.6% vs. LVI present 32.7%). Predicted and observed rates were similar for all categories, suggesting good model accuracy. Further details of the predicted probabilities and observed frequencies can be found in Table 3.

## 4. Discussion

In this study, we examined the critical relationship between tumor size, invasion into the subserosa and beyond, the presence of LVI, and the risk of regional node disease as a way to inform surgical management. Consistent with previous studies, we found that tumor size and LVI are associated with the presence of positive regional lymph nodes. However, contrary to current guidelines, invasion into the subserosa and beyond was not associated with an increased risk of nodal disease. Notably, in addition to tumor size, LVI remained a strong prognostic factor. In fact, the presence of LVI was associated with a high prevalence of nodal disease even in patients with tumors < 1 cm. To our knowledge, this is the first study to explore the association of high-risk pathologic features and nodal disease within this subgroup of patients for whom extensive surgical intervention remains controversial.

Our findings reaffirm the well-documented correlation between tumor size and nodal disease in ANETs. Although the risk is widely recognized [12,13,16], the appropriateness of current cutoffs for surgical decision-making might require reconsideration. Previous research advocating for more precise risk stratification identified 1 cm [16,17] and 1.55 cm [14] as more accurate thresholds for nodal disease risk; these cutoffs aligned with our observations of nearly 20% of patients with tumors between 1 and 2 cm exhibiting lymph node disease. Consistent with our results, lymphovascular invasion has repeatedly been shown to increase the risk of nodal disease [2,13,14,16], possibly elucidating why smaller size thresholds may be more predictive than the traditional 2 cm benchmark.

Other factors traditionally considered to increase the risk of nodal disease include tumor differentiation and tumor depth. Contrary to findings from other studies, our analysis did not reveal an independent association. The association between moderately differentiated tumors and nodal disease, initially observed in the univariate analysis, disappeared in the multivariate model, suggesting that this relationship may be influenced by other factors. Likewise, despite historical tendencies to recommend more aggressive surgical interventions based on tumor depth, our results, aligning with other well-powered studies, found no independent association with nodal disease [12,14]. Moreover, despite adapting our categorization of tumor depth (Table S3), in view that independent association with a more rigid definition of invasion has been observed [18], we observed no increased risk of nodal disease. This lack of correlation, even with refined invasion categories, challenges the reliability of using tumor depth alone for surgical decisions.

In this regard, the latest AJCC Cancer Staging Manual [15] uses tumor depth to define the primary tumor categories (T staging), suggesting that subserosal invasion carries a risk of nodal disease comparable to tumors >4 cm, as each feature alone, not in combination, is considered a T3 ANET. Yet, this is not the only place where this correlation is suggested; current guidelines consider tumor depth a “high-risk feature”, influencing discussions and considerations for right hemicolectomy even in tumors <1 cm [3,8,11]. All our analyses indicate that tumor depth does not independently predict nodal disease, proposing that historical correlation with nodal disease might be explained by the interplay between tumor depth and LVI. This aligns with early observations and recommendations by Bowman et al. [19] and Anderson et al. [20] from over four decades ago.

Given the profound implications, especially when tumor depth has been identified as the primary reason for extensive surgeries in centers of excellence [18], our findings add to the compelling need for a critical review of the surgical guidelines for ANETs.

Nevertheless, focusing on current recommendations, the question of when more extensive surgery, such as right hemicolectomy, is truly warranted remains unanswered. Holmager et al. [18] reported that 83% of patients undergoing right hemicolectomy did not have lymph node disease, underscoring the potential overtreatment based on estimated risk. Similarly, a recent meta-analysis [16] associated the completion of prophylactic right hemicolectomy with higher complication rates without clear clinical benefits. Regarding long-term outcomes, studies from national registries show no survival benefits for colectomy over appendectomy in ANETs [21,22]. Consequently, as suggested by Alabraba et al., selective surveillance and follow-up may be more appropriate than routine completion of right hemicolectomy [23].

While our study leverages a significant sample size, inherent limitations associated with using the NCDB such as potential coding and recording inaccuracies are part of our study [24]. To address the common issue of missing data, which often reduces the cohort size, we included all identified cases, regardless of missing information on key variables like tumor depth and lymphovascular invasion. However, to mitigate the impact of the missing data, we conducted a complete case analysis (Table S1), confirming that these results were consistent with our main findings. Similarly, other published papers in ANETs using national registries have underscored concerns regarding certain histological codes, as they can potentially capture more benign diseases; to address these concerns, our sensitivity analysis, focused on specific histology codes (8240 [carcinoid tumor] and 8241 [enterochromaffin cell carcinoid]), revealed no significant differences from our main findings. Likewise, since we calculate probabilities, considering potential confounders in lymph node sampling, we performed a sensitivity analysis including only patients with at least 12 evaluated nodes, which, similar to the rest of our sensitivity analysis, showed consistent results (Table S1).

Despite these analytical adjustments, we must acknowledge the exclusion of 11,638 cases due to insufficient nodal evaluation data. This exclusion likely introduces a selection bias towards cases with more comprehensive clinical documentation, possibly skewing towards more severe cases. This exclusion could potentially lead to an overestimation of the risk of nodal disease. However, this likely overestimation still supports our results and the broader discussion that surgical decisions in small tumors should not be based solely on individual factors such as tumor depth.

Our analysis was limited by the absence of detailed data such as precise tumor location, mesoappendiceal extension (expressed in mm), mitotic rate, Ki-67 proliferation rate, and additional information on more refined nuclear imaging modalities. Despite employing analytical approaches to bridge these gaps, we cannot ensure that our assumptions and surrogate data fully capture all possible interactions. Particular caution should be exercised when evaluating the impact of tumor grade, especially G3 tumors, on the risk estimation of nodal disease, as our analysis cannot estimate the actual effect of this feature due to current data limitations. We also recognize the potential existence of other molecular or disease markers not currently identified or recorded in the NCDB that could better inform the decision to perform surgery.

## 5. Conclusions

In conclusion, this study highlights the importance of considering a range of pathological features, alongside tumor size, in evaluating the risk of lymph node disease in ANETs. Our findings challenge the prevailing surgical management paradigm, which primarily focuses on tumor size and depth, by demonstrating the significant role of lymphovascular invasion in the risk of nodal disease. As such, for tumors smaller than 2 cm, when surgical resection is being considered, the extent of resection should not solely rely on tumor depth. Furthermore, we agree that the benefits of more extensive surgery, such as right hemicolectomy, in managing this slow-growing, primarily localized disease continue to warrant further exploration and discussion.

## Figures and Tables

**Table 1:** Patient characteristics.

	**LN (−)** **(n = 4349)**	**LN (+)** **(n = 1004)**	**Total** **(N = 5353)**	***p* ** **Univariate** **Analysis**
Baseline Characteristics				
Sex, n (%)				
Male	1725 (39.7)	357 (35.6)	2082 (38.9)	0.0161
Female	2624 (60.3)	647 (64.5)	3271 (61.1)	
Age (years), n (%)				
≤25	575 (13.2)	188 (18.7)	763 (14.3)	0.0001
26–50	1423 (32.7)	423 (42.1)	1846 (34.5)	
51–75	1931 (44.4)	353 (35.2)	2284 (42.7)	
≥76	420 (9.7)	40 (3.9)	460 (8.6)	
Race, n (%)				
Non-Hispanic White	3574 (82.2)	787 (78.4)	4361 (81.5)	0.0015
Non-Hispanic Black	347 (7.9)	123 (12.3)	470 (8.8)	
Hispanic	247 (5.7)	54 (5.4)	301 (5.6)	
Asian/Pacific Islander	84 (1.9)	19 (1.9)	103 (1.9)	
Other	62 (1.4)	11 (1.1)	73 (1.4)	
Unknown	35 (0.8)	10 (1)	45 (0.8)	
Charlson–Deyo Score, n (%)				
0–1	4028 (92.6)	970 (96.6)	4998 (93.4)	0.0001
≥2	321 (7.4)	34 (3.4)	355 (6.6)	
Insurance status, n (%)				
Not insured	143 (3.3)	39 (3.9)	182 (3.4)	<0.0001
Private	1702 (39.1)	262 (26.1)	1964 (36.7)	
Medicaid/Medicare/OG	2437 (56.1)	695 (69.2)	3132 (58.5)	
Unknown status	67 (1.5)	8 (0.8)	75 (1.4)	
Facility type, n (%)				
Community Cancer Program	191 (4.4)	57 (5.7)	248 (4.6)	<0.0001
Comprehensive Community Cancer	1203 (27.7)	204 (20.3)	1407 (26.3)	
Academic/Research Program	924 (21.3)	224 (22.3)	1148 (21.5)	
Integrated Network Cancer Program	679 (15.6)	115 (11.5)	794 (14.8)	
Unknown	1352 (31.1)	404 (40.2)	1756 (32.8)	
Pathological features				
Tumor differentiation, n (%)				
Well differentiated	3545 (81.5)	706 (70.3)	4251 (79.4)	<0.0001
Moderately differentiated	286 (6.6)	118 (11.8)	404 (7.6)	
Unknown	518 (11.9)	180 (17.9)	698 (13.1)	
LVI, n (%)				
Not present (−)	3241 (74.5)	369 (36.8)	3610 (67.4)	<0.0001
Present (+)	435 (10)	368 (36.7)	803 (15)	
Unknown	673 (15.5)	267 (26.6)	940 (17.6)	
Tumor depth, n (%)				
Confined to the appendiceal wall	818 (18.8)	234 (23.3)	1052 (19.7)	<0.0001
Invading the subserosa and beyond	855 (19.7)	247 (24.6)	1102 (20.6)	
Unknown	2676 (61.5)	523 (52.1)	3199 (59.8)	
Tumor size, n (%)				
<1 cm	2364 (54.4)	86 (8.6)	2450 (45.8)	<0.0001
≥1 cm but <2 cm	1138 (26.2)	257 (25.6)	1395 (26.1)	
≥2 cm	847 (19.5)	661 (65.8)	1508 (28.2)	
Number of sampled nodes, p50 (IQR)	16 (7–23)	20 (14–27)	16 (9–24)	<0.0001
Surgical margins, n (%)				
Negative margins	4230 (97.3)	917 (91.3)	5147 (96.2)	
Positive margins	68 (1.6)	62 (6.2)	130 (2.4)	
Unknown margins	51 (1.2)	25 (2.5)	76 (1.4)	

**Table 2:** Logistic regression models.

	Univariable			Multivariable		
Variables (Categories)	Odds Ratios	[95% Conf Interval]		Adjusted Odds Ratios	[95% Conf Interval]	
Sex	AUC = 0.52			AUC = 0.84		
Male	[Ref]			[Ref]		
Female	**1.19**	**1.03**	**1.37**	0.96	0.81	1.13
Age	AUC = 0.41					
≤25	[Ref]			[Ref]		
26–50	0.91	0.75	1.10	0.78	0.62	0.98
51–75	**0.56**	**0.46**	**0.68**	**0.68**	**0.54**	**0.87**
≥76	**0.29**	**0.20**	**0.42**	**0.54**	**0.35**	**0.85**
Race	AUC = 0.52					
Non-Hispanic White	[Ref]			[Ref]		
Non-Hispanic Black	**1.61**	**1.29**	**2.01**	1.23	0.95	1.60
Hispanic	0.99	0.73	1.35	0.76	0.54	1.09
Asian/Pacific Islander	1.03	0.62	1.70	1.29	0.71	2.32
Other	0.81	0.42	1.54	0.62	0.31	1.26
Charlson–Deyo Score	AUC = 0.48					
0–1	[Ref]			[Ref]		
≥2	**0.44**	**0.31**	**0.63**	0.74	0.49	1.12
Insurance status	AUC = 0.56					
Not insured	0.96	0.66	1.38	0.91	0.59	1.40
Medicaid/Medicare/OG	**0.54**	**0.46**	**0.63**	**0.78**	**0.64**	**0.95**
Private	[Ref]			[Ref]		
Unknown	0.42	0.20	0.88	0.39	0.17	0.88
Tumor differentiation	AUC = 0.56					
Well differentiated	[Ref]			[Ref]		
Moderately differentiated	**2.07**	**1.67**	**2.61**	1.25	0.96	1.64
Unknown	**1.74**	**1.44**	**2.11**	1.13	0.90	1.43
**LVI**	**AUC = 0.70**					
Lymph vascular (−)	[Ref]			[Ref]		
Lymph vascular (+)	**7.43**	**6.23**	**8.86**	**4.09**	**3.37**	**4.97**
Unknown	**3.48**	**2.92**	**4.16**	**2.42**	**1.95**	**3.01**
**Tumor depth**	AUC = 0.55					
Confined to the appendiceal wall	[Ref]			[Ref]		
Invading the subserosa and beyond	1.01	0.82	1.23	1.07	0.84	1.37
Unknown	0.68	0.57	0.81	0.79	0.64	0.97
**Tumor size**	AUC = 0.79					
<1 cm	[Ref]			[Ref]		
≥1 cm but <2 cm	**6.21**	**4.81**	**8.01**	**4.16**	**3.18**	**5.43**
≥2 cm	**21.45**	**16.91**	**27.22**	**14.43**	**11.27**	**18.49**
Surgical margins	AUC = 0.53					
Negative margins	[Ref]			[Ref]		
Positive margins	**4.21**	**2.96**	**5.98**	**2.52**	**1.67**	**3.89**
Unknown margins	**2.26**	**1.39**	**3.67**	**2.32**	**1.29**	**4.20**
All patients were included in the model (n = 5353).						

**Table 3:** Effect modification analysis and predicted probability of N+.

	Adjusted OR	Model Estimates	
	[95% CI]	Calculated Probability (95% PI), %	Observed Percentage, %
Tumor size <1 cm			
Confined to the appendiceal wall	[Ref]	2.2 (0.1–3.8)	2.1
Confined to the appendiceal wall + LVI	**10.1 [2.71–37.84]**	18.1 (2.3–3.4)	16.7
Invading the subserosa and beyond	1.39 [0.50–3.88]	2.9 (0.1–5.1)	2.9
Invading the subserosa and beyond + LVI	**14.1 [4.85–41.24]**	23.4 (10.1–36.8)	24.3
≥1 cm but <2 cm			
Confined to the appendiceal wall	[Ref]	13.9 (8.7–19.2)	14.6
Confined to the appendiceal wall + LVI	**5.86 [2.69–12.79]**	47.8 (32.2–63.3)	48.7
Invading the subserosa and beyond	0.73 [0.41–1.28]	10.6 (7.3–13.8)	10.8
Invading the subserosa and beyond + LVI	**3.07 [1.74–5.42]**	32.7 (25.2–40.3)	33.8
≥2 cm			
Confined to the appendiceal wall	[Ref]	30.1 (22.6–37.5)	31.7
Confined to the appendiceal wall + LVI	**3.85 [2.01–7.36]**	61.5 (49.1–73.9)	64.4
Invading the subserosa and beyond	1.54 [0.86–2.76]	39.6 (28.9–50.3)	41.1
Invading the subserosa and beyond + LVI	**5.20 [2.58–10.50]**	68.2 (55.4–81.1)	73.2

All patients were included in the model (n = 5353). This model was created using the same predictors as the ones included in Table 2 plus a two- and three-way term interaction between tumor size, tumor depth, and lymphovascular invasion; the coefficients for this model are presented in Table S3.

## Data Availability

The data used in the study are derived from a de-identified NCDB file. The American College of Surgeons and the Commission on Cancer have not verified and are not responsible for the analytic or statistical methodology employed, or the conclusions drawn from these data by the investigator. Restrictions apply to the availability of these data. The data were obtained through an application process by investigators associated with a Commission on Cancer-accredited cancer program. The data request process and additional information on this database can be reviewed at https://www.facs.org/quality-programs/cancer/ncdb/puf.

## Supplementary Materials

The following supporting information can be downloaded at: https://www.mdpi.com/article/10.3390/cancers16162922/s1, Table S1: Logistic regression models. [sensitivity analyses]. Table S2: Logistic regression model [sensitivity analysis]. Table S3: Multivariable interaction analysis.
