# Supplementary material for: Pathological Features Associated with Lymph Node Disease in Patients with Appendiceal Neuroendocrine Tumors
Source: Cancers (Basel). 2024 Aug 22;16(16):2922. doi: 10.3390/cancers16162922 (PMC11352421; doi:10.3390/cancers16162922)
Supplement: Supplementary file 1 [file cancers-16-02922-s001.zip › cancers-3142769-supplementary.pdf]

Supplementary materials.

Table S1. Logistic regression models.

Model	Complete case analysis n = 1492	Limited histology n = 4478	≥12 nodes evaluated n = 3776	Combined n=845
AUC	AUC= 0.84	AUC = 0.85	AUC = 0.83	AUC = 0.82
Variables (categories)	Odds ratios [95% CI]	Odds ratios [95% CI]	Odds ratios [95% CI]	Odds ratios [95% CI]
Sex				
Male	[Ref]	[Ref]	[Ref]	[Ref]
Female	0.8 [0.6-1.1]	0.9 [0.8-1.2]	0.9 [0.8-1.1]	0.9 [0.6-1.4]
Age				
≤ 25	[Ref]	[Ref]	[Ref]	[Ref]
26 – 50	**0.7 [0.4-0.9]**	0.8 [0.6-1.1]	**0.7 [0.6-0.9]**	0.7 [0.4-1.1]
51 – 75	**0.5 [0.3-0.8]**	**0.7 [0.5-0.9]**	**0.6 [0.5-0.8]**	**0.5 [0.3-0.9]**
≥ 76	0.4 [0.2-1.1]	**0.7 [0.4-0.9]**	**0.5 [0.3-0.8]**	0.4 [0.1-1.3]
Race				
Non-Hispanic white	[Ref]	[Ref]	[Ref]	[Ref]
Non-Hispanic black	**2.2 [1.4-3.4]**	1.1 [0.8-1.5]	1.4 [1.1-1.9]	1.7 [0.9-3.4]
Hispanic	0.8 [0.4-1.5]	0.7 [0.5-1.1]	0.8 [0.5-1.2]	0.7 [0.3-1.7]
Asian/Pacific Islander	0.8 [0.2-3.2]	1.5 [0.8-2.9]	1.1 [0.5-2.0]	0.7 [0.1-3.6]
Other	0.4 [0.2-4.1]	0.7 [0.6-3.5]	0.7 [0.4-1.5]	0.6 [0.1-2.8]
Charlson-Deyo Score				
0-1	[Ref]	[Ref]	[Ref]	[Ref]
≥2	0.9 [0.4-2.3]	0.9 [0.5-1.4]	0.7 [0.4-1.1]	0.9 [0.3-2.8]
Insurance Status				
Not insured	0.5 [0.2-1.2]	0.8 [0.5-1.4]	0.8 [0.5-1.3]	**0.3 [0.0-0.9]**
Medicaid/Medicare/OG	**0.7 [0.5-0.9]**	**0.7 [0.6-0.9]**	**0.8 [0.6-0.9]**	0.7 [0.4-1.2]
Private	[Ref]	[Ref]	[Ref]	[Ref]
Tumor Differentiation				
Well-differentiated	[Ref]	[Ref]	[Ref]	[Ref]
Moderately differentiated	1.18 [0.7-1.9]	1.43 [0.7-1.9]	1.1 [0.8-1.5]	1.0 [0.5-2.2]
Unknown		1.1 [0.9-1.5]	1.2 [0.9-1.5]	[excluded]
**<u>LVI</u>**				
Lymph Vascular (−)	[Ref]	[Ref]	[Ref]	[Ref]
Lymph Vascular (+)	**4.8 [3.5-6.7]**	**4.0 [3.2-4.9]**	**3.7 [3.0-4.6]**	**4.4 [2.9-6.5]**
Unknown	[excluded]	**2.6 [2.1-3.3]**	**2.4 [1.8-2.9]**	[excluded]
**<u>Tumor depth</u>**				
Confined to the appendiceal wall	[Ref]	[Ref]	[Ref]	[Ref]
Invading the subserosa and beyond	0.97 [0.7-1.4]	1.1 [0.8-1.5]	1.0 [0.8-1.3]	1.1 [0.7-1.8]
Unknown	[excluded]	**0.7 [0.6-0.9]**	0.8 [0.6-1.1]	[excluded]
**<u>Tumor size</u>**				
<1 cm	[Ref]	[Ref]	[Ref]	[Ref]
≥1 cm but <2 cm	**3.6 [2.3-5.8]**	**4.1 [3.1-5.6]**	**3.6 [2.7-4.9]**	**3.0 [1.7-5.2]**
≥2 cm	**13.2 [8.1-21.5]**	**14.7 [11.1-19.4]**	**11.8 [8.9-15.6]**	**9.3 [5.0-17.4]**
Surgical Margins				
Negative margins	[Ref]	[Ref]	[Ref]	[Ref]
Positive margins	1.9 [0.9-4.1]	**3.0 [1.9-4.8]**	**2.5 [1.5-4.2]**	1.9 [0.7-5.8]
Unknown margins	[excluded]	1.6 [0.8-3.2]	**2.2 [1.0-4.3]**	[excluded]**]**

Table S2. Logistic regression models.

Variables (categories)	Odds ratios [95% CI]<br>Expanding categories for extension<br>N = 5353
Sex	
Male	[Ref]
Female	0.9 [0.8-1.1]
Age	
≤ 25	[Ref]
26 – 50	**0.8 [0.6-0.9]**
51 – 75	**0.7 [0.5-0.9]**
≥ 76	**0.5 [0.3-0.8]**
Race	
Non-Hispanic white	[Ref]
Non-Hispanic black	1.2 [0.9-1.6]
Hispanic	0.8 [0.5-1.1]
Asian/Pacific Islander	1.3 [0.7-2.3]
Other	0.6 [0.3-1.2]
Charlson-Deyo Score	
0-1	[Ref]
≥2	0.7 [0.5-1.3]
Insurance Status	
Not insured	0.9 [0.6-1.4]
Medicaid/Medicare/OG	**0.7 [0.6-0.9]**
Private	[Ref]
Tumor Differentiation	
Well-differentiated	[Ref]
Moderately differentiated	1.3 [0.9-1.6]
Unknown	1.12 [0.8-1.4]
**LVI**	
Lymph Vascular (−)	[Ref]
Lymph Vascular (+)	**4.1 [3.4-4.9]**
Unknown	**2.4 [1.9-3.0]**
**Tumor depth**	
Mucosa and submucosa	[Ref]
Infiltration of mesoappendix	0.9 [0.7-1.2]
Extended beyond than mesoappendix	1.4 [0.9-2.0]
Unknown	0.8 [0.6-0.9]
**Tumor size**	
<1 cm	[Ref]
≥1 cm but <2 cm	**4.1 [3.2-4.9]**
≥2 cm	**14.3 [11.1-18.3]**
Surgical Margins	
Negative margins	[Ref]
Positive margins	**2.5 [1.6-3.8]**
Unknown margins	**2.3 [1.3-4.1]**

Table S3. Multivariable interaction analysis			
	Multivariable		
Variables (categories)	Odds ratios	[95% Conf Interval]	
Sex			
Male	[Ref]		
Female	0.96	0.81	1.13
Age			
≤ 25	[Ref]		
26 – 50	**0.78**	**0.62**	**0.98**
51 – 75	**0.70**	**0.55**	**0.88**
≥ 76	**0.56**	**0.36**	**0.87**
Race			
Non-Hispanic white	[Ref]		
Non-Hispanic black	1.21	0.93	1.57
Hispanic	0.77	0.54	1.09
Asian/Pacific Islander	1.30	0.72	2.36
Other	0.61	0.30	1.23
Charlson-Deyo Score			
0-1	[Ref]		
≥2	0.77	0.50	1.16
Insurance Status			
Not insured	0.93	0.60	1.44
Medicaid/Medicare/OG	0.78	0.64	0.95
Private	[Ref]		
Unknown	0.39	0.17	0.88
Tumor Differentiation			
Well-differentiated	[Ref]		
Moderately differentiated	1.23	0.94	1.61
Unknown	1.11	0.87	1.40
**Tumor depth**			
Confined to the appendiceal wall	[Ref]		
Invading the subserosa and beyond	1.39	1.39	1.39
	0.81	0.81	0.81
**Tumor size**			
<1 cm	[Ref]		
≥1 cm but <2 cm	4.16	4.16	4.16
≥2 cm	14.43	14.43	14.43
**Tumor size* Tumor depth**			
≥1 cm but <2 cm* Invading the subserosa and beyond	0.52	0.16	1.70
≥1 cm but <2 cm* Unknown	0.80	0.28	2.24
≥2 cm* Invading the subserosa and beyond	1.12	0.34	3.64
≥2 cm* Unknown	1.20	0.46	3.13
**LVI**			
Lymph Vascular (-)	[Ref]		
Lymph Vascular (+)	10.14	2.71	37.84
Unknown	3.20	1.09	9.42
**Tumor size* LVI**			
≥1 cm but <2 cm* Lymph Vascular (+)	0.58	0.13	2.67
≥1 cm but <2 cm* Unknown	1.07	0.30	3.79
≥2 cm* Lymph Vascular (+)	0.38	0.09	1.65
≥2 cm* Unknown	0.61	0.19	1.99
**Tumor depth* LVI**			
Invading the subserosa and beyond* Lymph Vascular (+)	1.01	0.19	5.38

Invading the subserosa and beyond* Unknown	1.37	0.26	7.27
Unknown* Lymph Vascular (+)	1.35	0.29	6.36
Unknown * Unknown	1.31	0.37	4.64
**Tumor size* Tumor depth* LVI**			
≥1 cm but <2 cm* Invading the subserosa and beyond* LVI(+)	0.72	0.11	4.84
≥1 cm but <2 cm* Invading the subserosa and beyond* Unknown	0.57	0.08	3.86
≥1 cm but <2 cm* Unknown * LVI(+)	0.44	0.07	2.75
≥1 cm but <2 cm* Unknown * Unknown	0.61	0.13	2.89
≥2 cm* Invading the subserosa and beyond* LVI(+)	0.87	0.12	6.09
≥2 cm* Invading the subserosa and beyond* Unknown	0.82	0.13	5.26
≥2 cm* Unknown * LVI(+)	0.62	0.11	3.43
≥2 cm* Unknown * Unknown	0.71	0.17	2.95
Surgical Margins			
Negative margins	[Ref]		
Positive margins	**2.52**	**2.52**	**2.52**
Unknown margins	**2.32**	**2.32**	**2.32**

All patients were included in the model (N=5353).
